# Case Analysis of a Patient with Functional Pathological Crying

**DOI:** 10.1192/j.eurpsy.2024.1476

**Published:** 2024-08-27

**Authors:** T. S. A. Tay

**Affiliations:** Psychological Medicine, Changi General Hospital, Singapore, Singapore

## Abstract

**Introduction:**

Functional pathological crying is a complex psychic phenomenon which poses both diagnostic and management challenges to the psychiatrist and psychotherapist. Apart from treatment with medications when clinically indicated, psychodynamic psychotherapy can be useful to understand the aetiology and to address these psychological issues faced by patients.

**Objectives:**

In this case report, psychodynamic psychotherapeutic techniques are employed to examine and manage functional pathological crying.

Ms L was a 33-year-old Chinese single woman who presented with mixed depressive and anxiety symptoms associated with frequent severe bouts of wailing. She had a history of parental neglect and childhood sexual abuse. Following psychiatric assessment, she was diagnosed with Mixed Depressive and Anxiety Disorder, and Borderline Personality Disorder. She was treated with Sertraline 50mg every morning and was referred for psychodynamic therapy.

**Methods:**

Building trust and rapport with Ms L was crucial so that the therapeutic relationship could be utilized as a vehicle for change through earned attachments. Helping her appreciate how present experiences reflect conflicts from her past and addressing her defence mechanisms with the aims of expression of emotions, exploring her wishes and fantasies to access unconscious conflicts were important. These build greater self-awareness which helped her to develop the capacity for emotional self-regulation, bringing about an increase in her level of adaptation to stressors.

**Results:**

During the early phase of therapy, Ms L would be wailing throughout most of the therapy hour. As therapeutic rapport and trust were established, she began to open up about her abuse for us to explore her conflicts and complex emotions associated with it.

The key themes that emerged were her chronic low self-esteem with fears of authorities and abandonment, the tendency to take up a defended regressive helpless child-like position whenever feelings related to the abuse were rekindled, as well as the manifestation of these complicated psychic experiences in the form of a complex wailing phenomenon.

The functional pathological crying was a mixture of an expression of her challenging conflictual painful feelings, symbolic expression of her cry for help, repressive and regressive child-like emotional states as well as having a defensive function to avoid coming in touch with painful feelings.

Through therapy, Ms L was able to make better sense of her wailing, develop the capacity for emotional self-regulation by adopting a healthier adult position in responding to difficult feelings when triggered, learning to forgive herself and others, assume better self-care and improved relationships with others.

**Conclusions:**

Through psychodynamic psychotherapy, complex functional pathological crying can be better understood and managed to bring about intra-psychic and interpersonal functional improvements.

**Disclosure of Interest:**

None Declared

